# Supplementary material for: Seroprevalence of antibodies against SARS-CoV-2 among health care workers in a large Spanish reference hospital
Source: Nat Commun. 2020 Jul 8;11:3500. doi: 10.1038/s41467-020-17318-x (PMC7343863; doi:10.1038/s41467-020-17318-x)
Supplement: Supplementary file 1 — Supplementary Information [file 41467_2020_17318_MOESM1_ESM.pdf]

Supplementary Information

Supplementary Note 1

We used primers from the US CDC panel:

https://www.cdc.gov/coronavirus/2019-ncov/lab/rt-pcr-panel-primer-probes.html

Sequences are:
N1:
2019-nCoV_N1-F GAC CCC AAA ATC AGC GAA AT 500 nM final concentration
2019-nCoV_N1-R TCT GGT TAC TGC CAG TTG AAT CTG 500 nM final concentration
2019-nCoV_N1-P FAM-ACC CCG CAT /ZEN/ TAC GTT TGG TGG ACC-3IABkFQ 125 nM final concentration

N2:
2019-nCoV_N2-F TTA CAA ACA TTG GCC GCA AA 500 nM final concentration
2019-nCoV_N2-R GCG CGA CAT TCC GAA GAA 500 nM final concentration
2019-nCoV_N2-P FAM-ACA ATT TGC /ZEN/ CCC CAG CGC TTC AG-3IABkF 125 nM final concentration

RNaseP
RP-F AGA TTT GGA CCT GCG AGC G 500 nM final concentration
RP-R GAG CGG CTG TCT CCA CAA GT 500 nM final concentration
RP-P FAM-TTC TGA CCT /ZEN/ GAA GGC TCT GCG CG-3IABkFQ 125 nM final concentration

Supplementary Table 1. Baseline characteristics of study participants stratified by age group.

		<45y (n=314)	>=45y (n=264)	Total	P-value
**Sex** [1]	*Male*	88 (28%)	73 (28%)	161 (28%)	0.9204 [2]
	Female	226 (72%)	191 (72%)	417 (72%)	
**Professional category** [1]	*Nurse / Auxiliary nurse / Stretcher-bearer*	166 (53%)	122 (46%)	288 (50%)	0.0020 [2]
	Physician	86 (27%)	61 (23%)	147 (25%)	
	Lab technologist / other tech.	26 (8%)	19 (7%)	45 (8%)	
	Administrative officers, Other[6]	36 (11%)	62 (23%)	98 (17%)	
**Age** [3]		33 (6.6)	52,9 (5.3)	42,1 (11.6)	
**Daily contact with patients**	*No*	55 (18%)	68 (26%)	123 (21%)	0.0159 [2]
	Yes	259(82%)	196 (74%)	455 (79%)	
**Working in a COVID19 unit** [1]	*No*	151 (48%)	164 (62%)	315 (54%)	0.0007 [2]
	Yes	163 (52%)	100 (38%)	263 (46%)	
**Close contact with COVID19 confirmed or suspected case** [1]	*No*	65 (21%)	72 (27%)	137 (24%)	0.0642 [2]
	Yes	249 (79%)	192 (73%)	441 (76%)	
**Previously diagnosed with COVID19 by rRT-PCR**[1]	*No*	292 (93%)	247 (94%)	539 (93%)	0.7866 [2]
	Yes	22 (7%)	17 (6%)	39 (7%)	
**Comorbidities**[1]*****	*No*	292 (93%)	225 (85%)	517 (89%)	0.0025 [2]
	Yes	22 (7%)	39 (15%)	61 (11%)	
**Household size** [3]		2.6 (1.1)	2.9 (1.2)	2.8 (1.2)	0.0200 [4]
**Received Flu vaccine (2019-2020 season)** [1]	*No*	175 (56%)	164 (62%)	339 (59%)	0.1203 [2]
	Yes	139 (44%)	100 (38%)	239 (41%)	
**Reporting COVID-19 compatible symptoms within previous month** [1]	*No*	196 (62%)	172 (65%)	368 (64%)	0.4965 [2]
	Yes	118 (38%)	92 (35%)	210 (36%)	

1: n (Column percentage)
2: Chi-squared test
3: Arithmetic Mean (SD) [n]
4: t-test
5: Fisher's exact test
6. Includes, cleaning, kitchen and maintenance staff
* Comorbidities include: heart and liver disease, diabetes, chronic respiratory and renal disease, cancers and autoimmune and other immunological disorders.

Supplementary Table 2. Serology results in samples from participants previously diagnosed of COVID-19 by rRT-PCR and in samples from pre-pandemic negative controls

Combination	rRT-PCR+/ Sero−	Pre-pandemic/ Sero+	Pre-pandemic/ Sero−	rRT-PCR+/ Sero+	Total	Sensitivity (%)	Specificity (%)	PPV (%)	NPV (%)
IgA RBD	6	1	46	33	86	85	98	97	88
IgA RBD &/or IgG RBD	6	1	46	33	86	85	98	97	88
IgA RBD &/or IgG RBD &/or IgM RBD	6	1	46	33	86	85	98	97	88
IgA RBD &/or IgM RBD	6	1	46	33	86	85	98	97	88
IgG RBD	7	0	47	32	86	82	100	100	87
IgG RBD &/or IgM RBD	7	0	47	32	86	82	100	100	87
IgM RBD	14	0	47	25	86	64	100	100	77

N= 39 participants with positive past rRT-PCR samples and 47 pre-pandemic negative controls. PPV, positive predictive value; NPV, negative predictive value.

Supplementary Table 3. Seroprevalence of different antibodies (and combination of) stratified by days since onset of symptoms.

Combination	Days since onset of symptoms	Sero-	Sero+	Total	Sero+ (%)
IgA RBD	1-9 days	31	2	33	6.06
IgA RBD	10-19 days	47	20	67	29.85
IgA RBD	20-29 days	47	16	63	25.4
IgA RBD	30-39 days	31	2	33	6.06
IgA RBD	40-49 days	7	1	8	12.5
IgA RBD	50-59 days	5	0	5	0
IgA RBD	60-69 days	1	0	1	0
IgA RBD	No symptoms	362	6	368	1.63
IgA RBD &/or IgG RBD	1-9 days	31	2	33	6.06
IgA RBD &/or IgG RBD	10-19 days	46	21	67	31.34
IgA RBD &/or IgG RBD	20-29 days	47	16	63	25.4
IgA RBD &/or IgG RBD	30-39 days	31	2	33	6.06
IgA RBD &/or IgG RBD	40-49 days	7	1	8	12.5
IgA RBD &/or IgG RBD	50-59 days	5	0	5	0
IgA RBD &/or IgG RBD	60-69 days	1	0	1	0
IgA RBD &/or IgG RBD	No symptoms	360	8	368	2.17
IgA RBD &/or IgG RBD &/or IgM RBD	1-9 days	31	2	33	6.06
IgA RBD &/or IgG RBD &/or IgM RBD	10-19 days	45	22	67	32.84
IgA RBD &/or IgG RBD &/or IgM RBD	20-29 days	47	16	63	25.4
IgA RBD &/or IgG RBD &/or IgM RBD	30-39 days	31	2	33	6.06
IgA RBD &/or IgG RBD &/or IgM RBD	40-49 days	7	1	8	12.5
IgA RBD &/or IgG RBD &/or IgM RBD	50-59 days	5	0	5	0
IgA RBD &/or IgG RBD &/or IgM RBD	60-69 days	1	0	1	0
IgA RBD &/or IgG RBD &/or IgM RBD	No symptoms	357	11	368	2.99
IgA RBD &/or IgM RBD	1-9 days	31	2	33	6.06
IgA RBD &/or IgM RBD	10-19 days	46	21	67	31.34
IgA RBD &/or IgM RBD	20-29 days	47	16	63	25.4
IgA RBD &/or IgM RBD	30-39 days	31	2	33	6.06
IgA RBD &/or IgM RBD	40-49 days	7	1	8	12.5
IgA RBD &/or IgM RBD	50-59 days	5	0	5	0
IgA RBD &/or IgM RBD	60-69 days	1	0	1	0
IgA RBD &/or IgM RBD	No symptoms	358	10	368	2.72
IgG RBD	1-9 days	32	1	33	3.03
IgG RBD	10-19 days	47	20	67	29.85
IgG RBD	20-29 days	47	16	63	25.4
IgG RBD	30-39 days	31	2	33	6.06
IgG RBD	40-49 days	7	1	8	12.5
IgG RBD	50-59 days	5	0	5	0
IgG RBD	60-69 days	1	0	1	0
IgG RBD	No symptoms	364	4	368	1.09

IgG RBD &/or IgM RBD	1-9 days	32	1	33	3.03
IgG RBD &/or IgM RBD	10-19 days	45	22	67	32.84
IgG RBD &/or IgM RBD	20-29 days	47	16	63	25.4
IgG RBD &/or IgM RBD	30-39 days	31	2	33	6.06
IgG RBD &/or IgM RBD	40-49 days	7	1	8	12.5
IgG RBD &/or IgM RBD	50-59 days	5	0	5	0
IgG RBD &/or IgM RBD	60-69 days	1	0	1	0
IgG RBD &/or IgM RBD	No symptoms	361	7	368	1.9
IgM RBD	1-9 days	32	1	33	3.03
IgM RBD	10-19 days	51	16	67	23.88
IgM RBD	20-29 days	52	11	63	17.46
IgM RBD	30-39 days	31	2	33	6.06
IgM RBD	40-49 days	7	1	8	12.5
IgM RBD	50-59 days	5	0	5	0
IgM RBD	60-69 days	1	0	1	0
IgM RBD	No symptoms	363	5	368	1.36

Supplementary Table 4. Serology results in samples from participants previously diagnosed of COVID-19 by rRT-PCR and more than 10 days since onset of symptoms and pre-pandemic negative controls

Combination	rRT-PCR+/ Sero− [a]	Pre-pandemic/ Sero+	Pre-pandemic/ Sero−	rRT-PCR+/ Sero+	Total	Sensitivity (%)	Specificity (%)	PPV (%)	NPV (%)
**IgG RBD**	1	0	47	31	79	97	100	100	98
**IgG RBD &/or IgM RBD**	1	0	47	31	79	97	100	100	98
**IgA RBD**	1	1	46	31	79	97	98	97	98
**IgA RBD &/or IgG RBD**	1	1	46	31	79	97	98	97	98
**IgA RBD &/or IgG RBD &/or IgM RBD**	1	1	46	31	79	97	98	97	98
**IgA RBD &/or IgM RBD**	1	1	46	31	79	97	98	97	98
**IgM RBD**	8	0	47	24	79	75	100	100	85

[a] The onset of symptoms of the seronegative participant with positive past rRT-PCR was 19 days before serology testing.

N= 32 participants with positive past rRT-PCR results and 47 pre-pandemic negative controls. PPV, positive predictive value; NPV, negative predictive value.

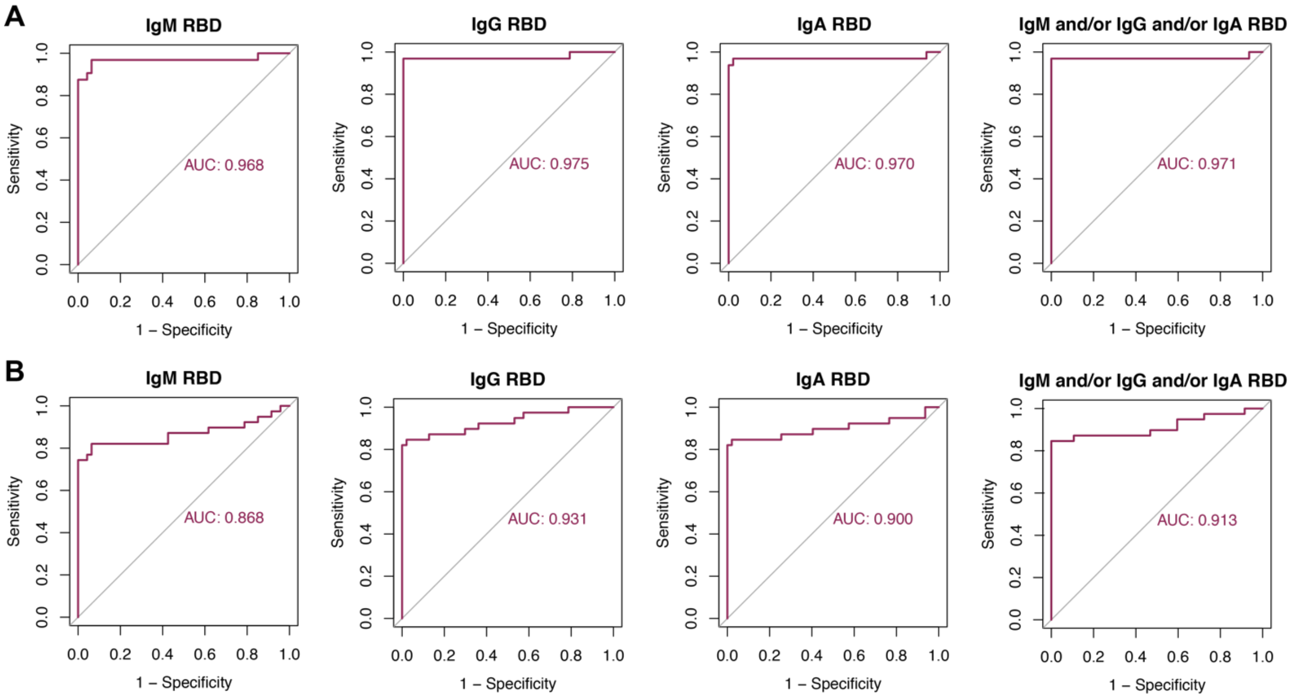

Supplementary Figure 1. Antibody Luminex assay performance. Receiver operating characteristic (ROC) curve and area under the ROC curve (AUC) for each antibody isotype and the combination of the three isotypes using samples from participants with positive past rRT-PCR and more than 10 days since onset of symptoms (A), and samples from all participants with positive past rRT-PCR regardless of time since onset of symptoms (B). Samples from 32 (A) and 39 (B) study participants and 47 pre-pandemic negative controls were used.

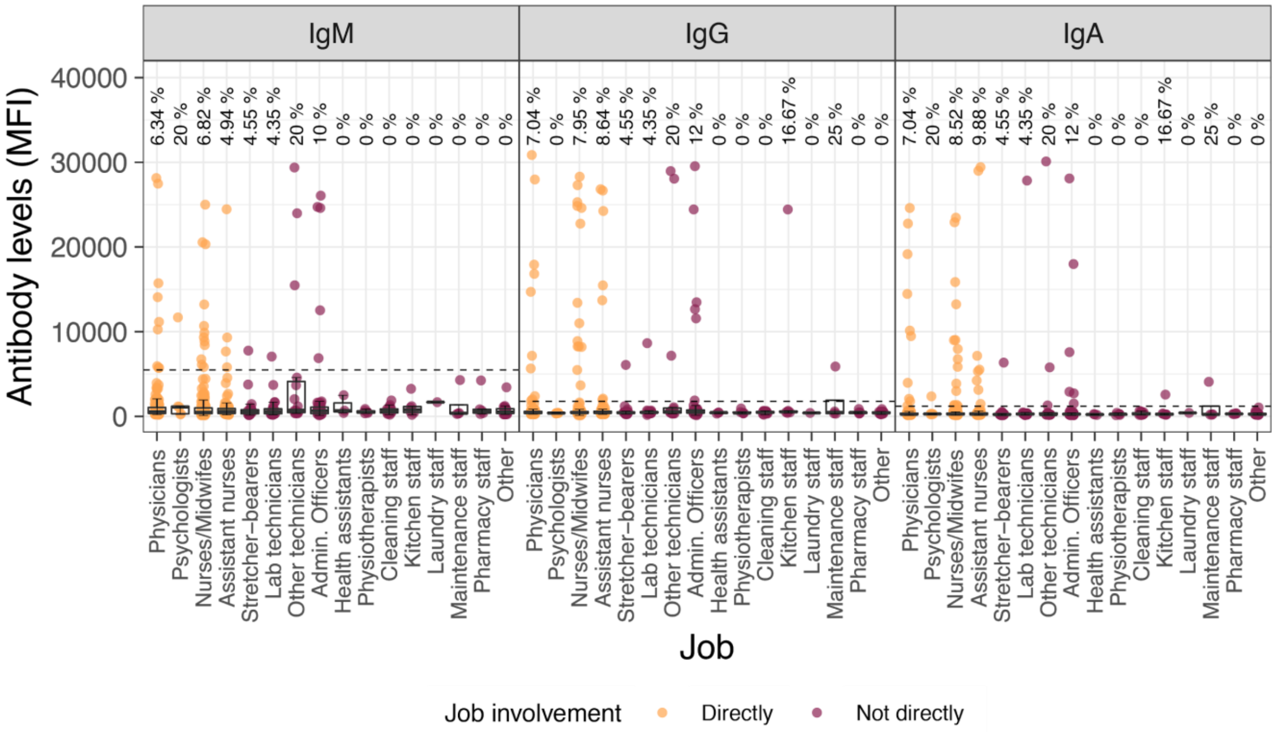

Supplementary Figure 2. Antibody levels and seroprevalence by professional category. Levels (median fluorescence intensity, MFI) of IgM, IgG and IgA against Receptor Binding Domain of the SARS-CoV-2 Spike glycoprotein by professional role. The dashed line marks the seropositivity threshold. Orange and burgundy dots show subjects working directly with COVID-19 patients or not working directly or not directly with patients, respectively.
