# Supplementary material for: Seroprevalence of antibodies against SARS-CoV-2 among health care workers in a large Spanish reference hospital
Source: Nat Commun. 2020 Jul 8;11:3500. doi: 10.1038/s41467-020-17318-x (PMC7343863; doi:10.1038/s41467-020-17318-x)
Supplement: Supplementary file 2 — Peer Review File [file 41467_2020_17318_MOESM2_ESM.pdf]

Editorial Note:

This manuscript has been previously reviewed at another journal that is not operating a transparent peer review scheme. The manuscript was considered suitable for publication without further review at *Nature Communications*.
